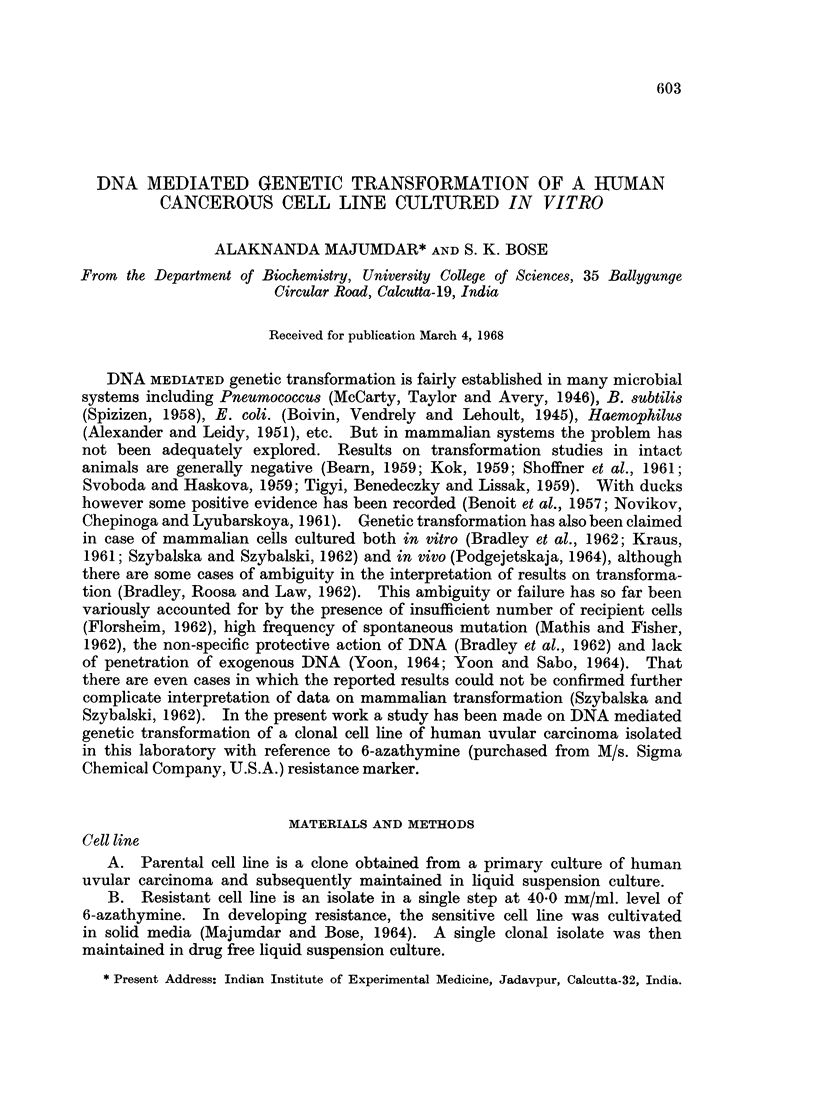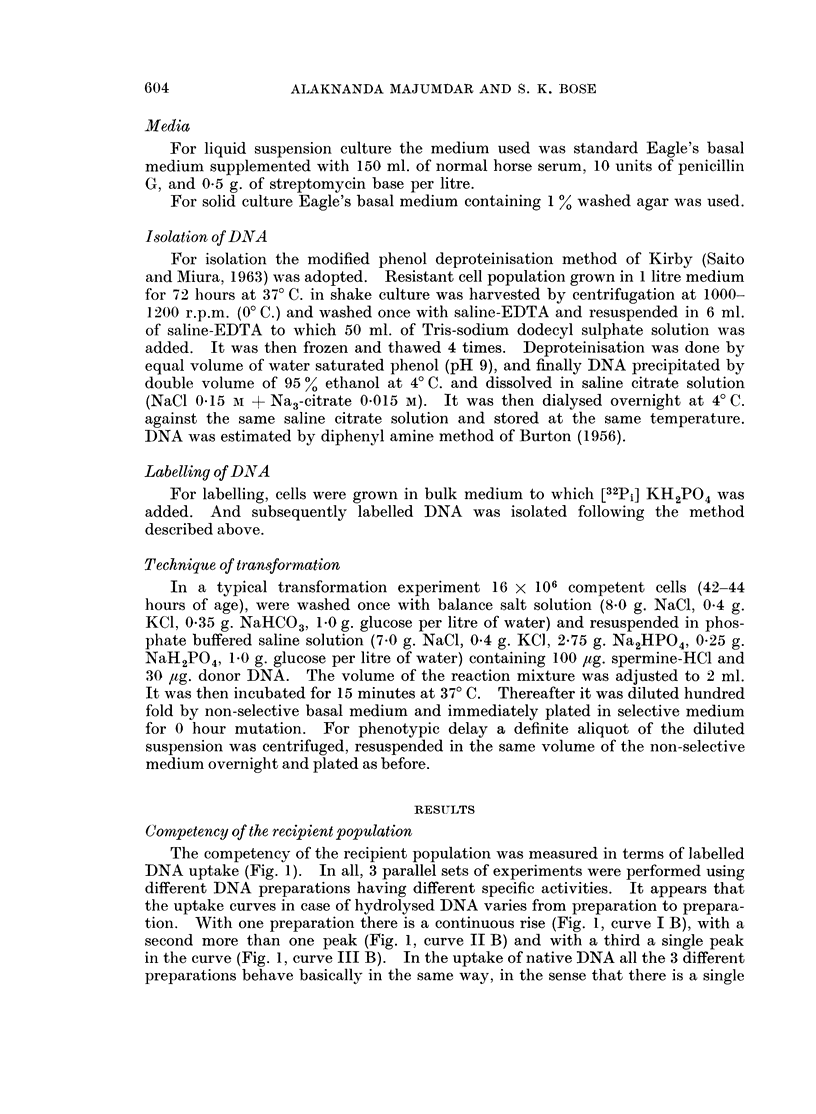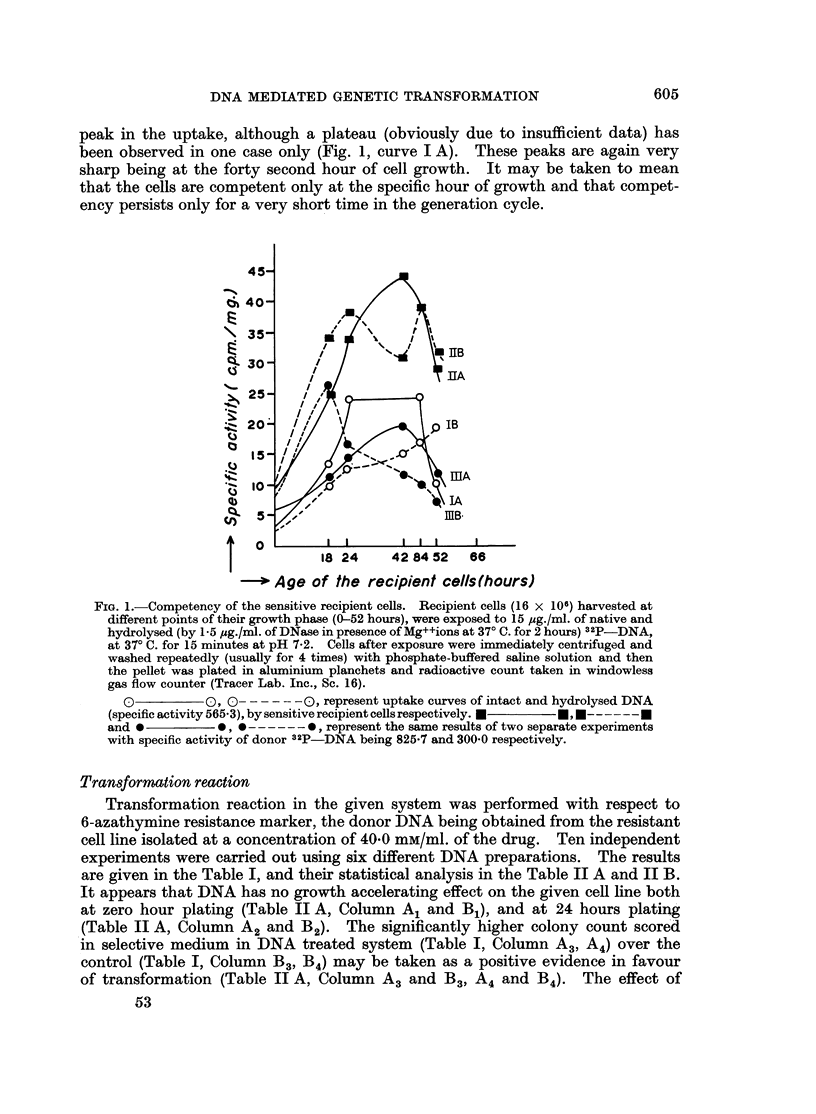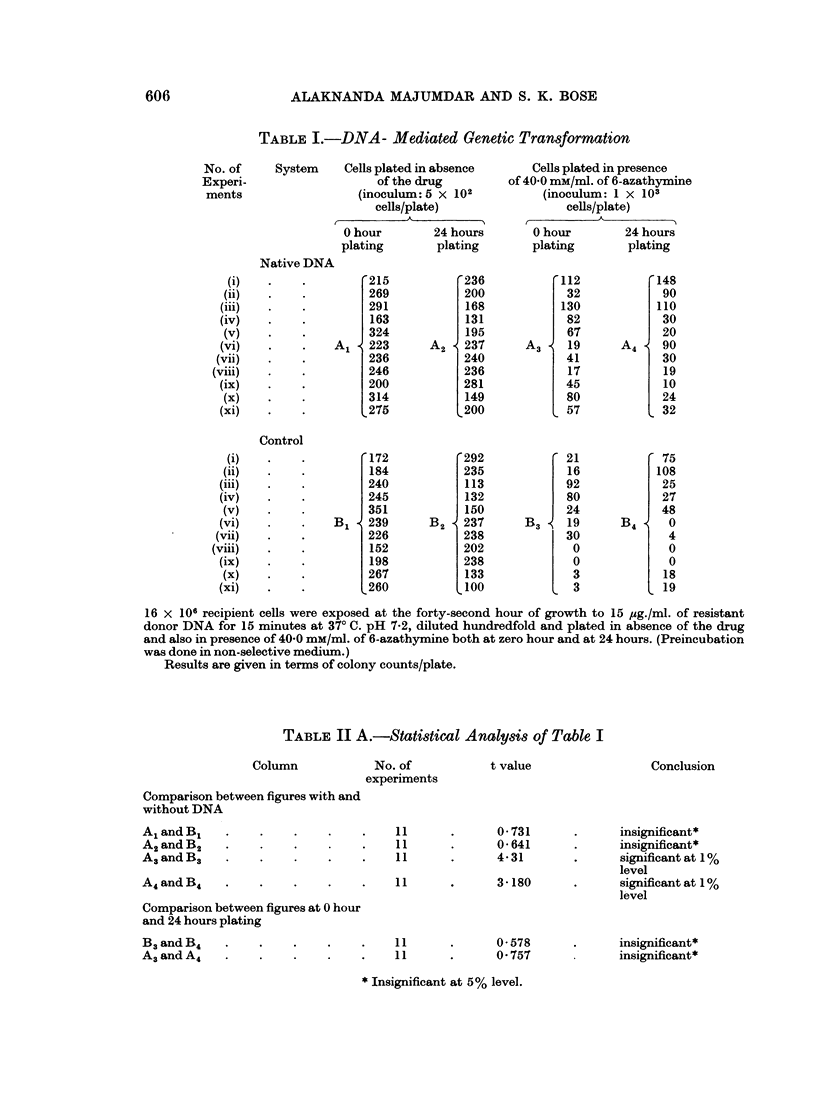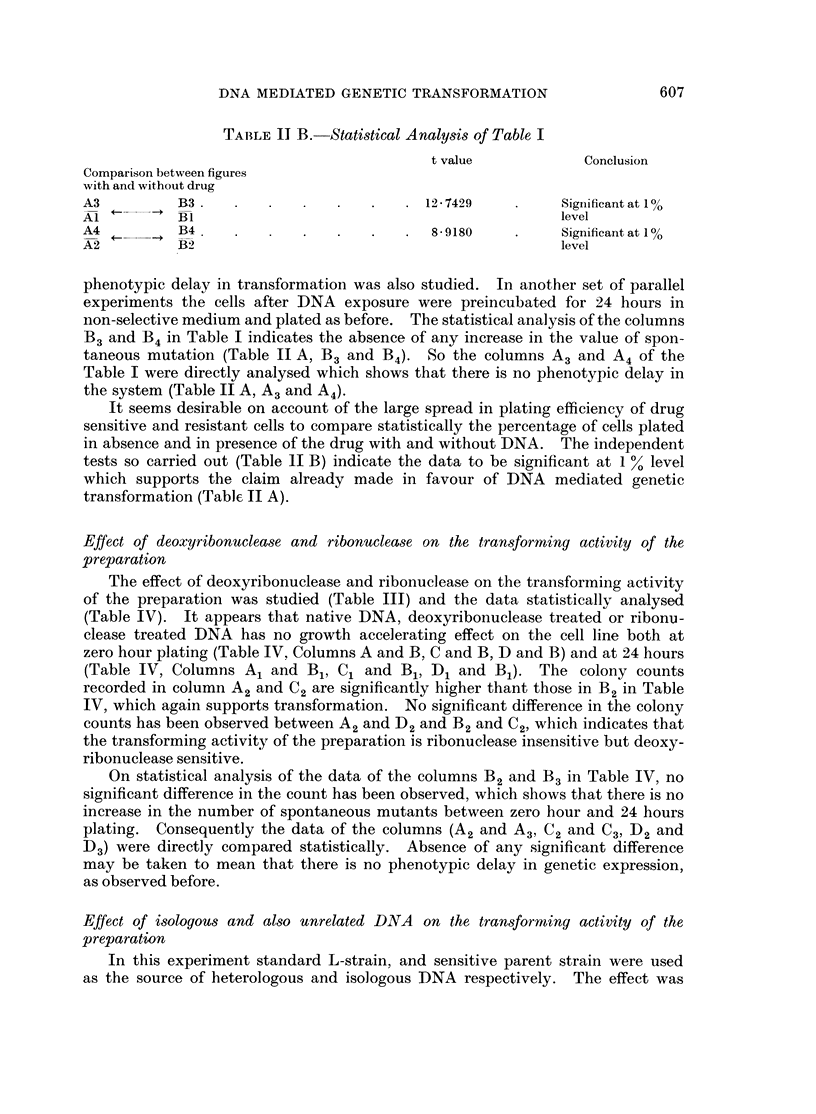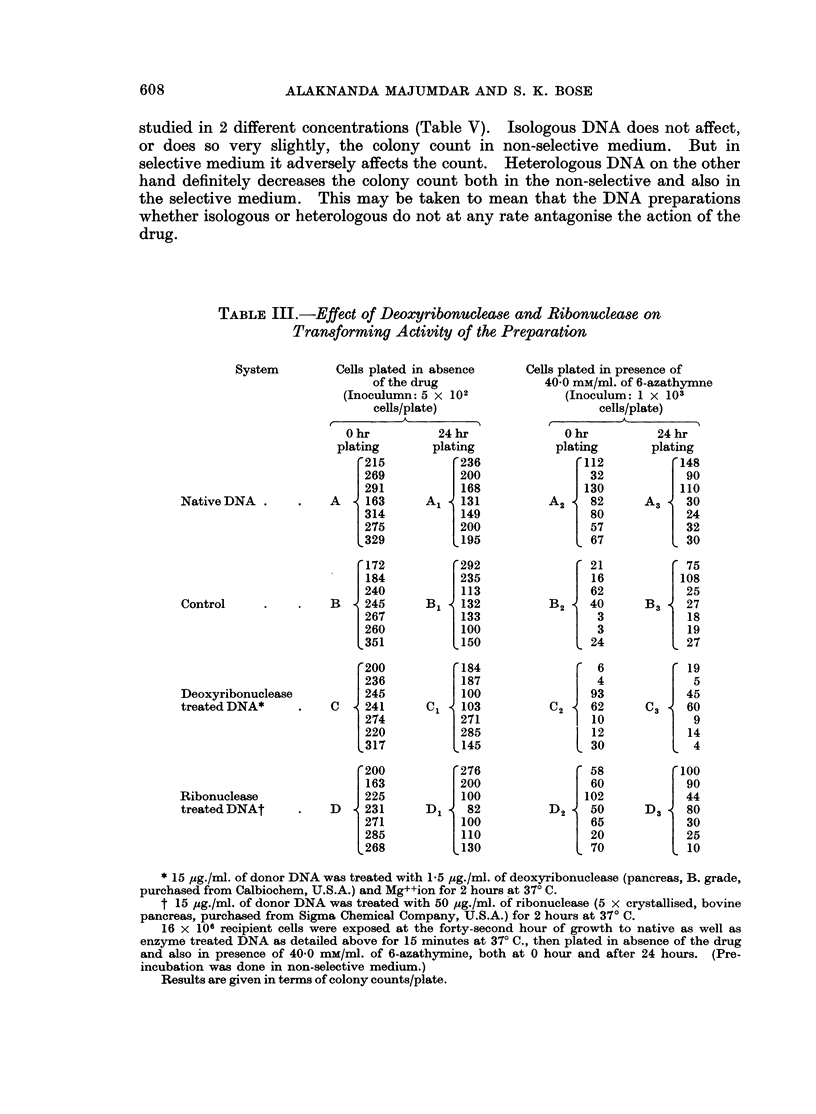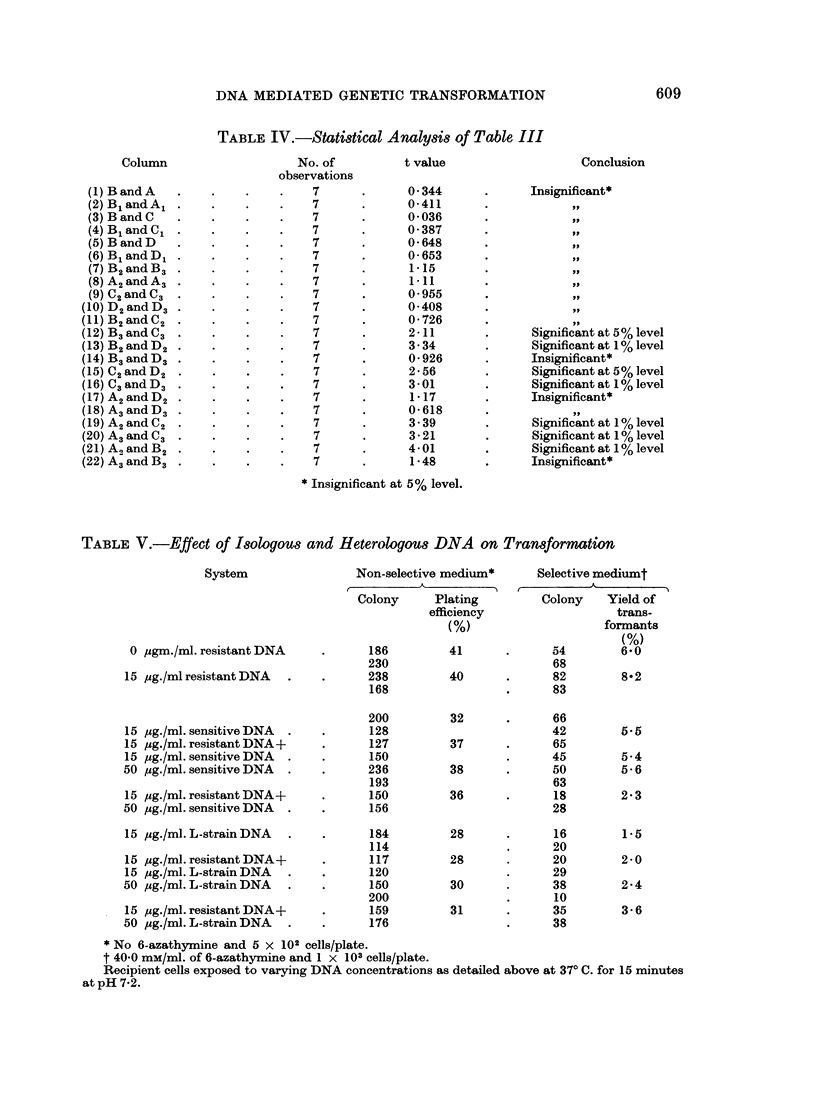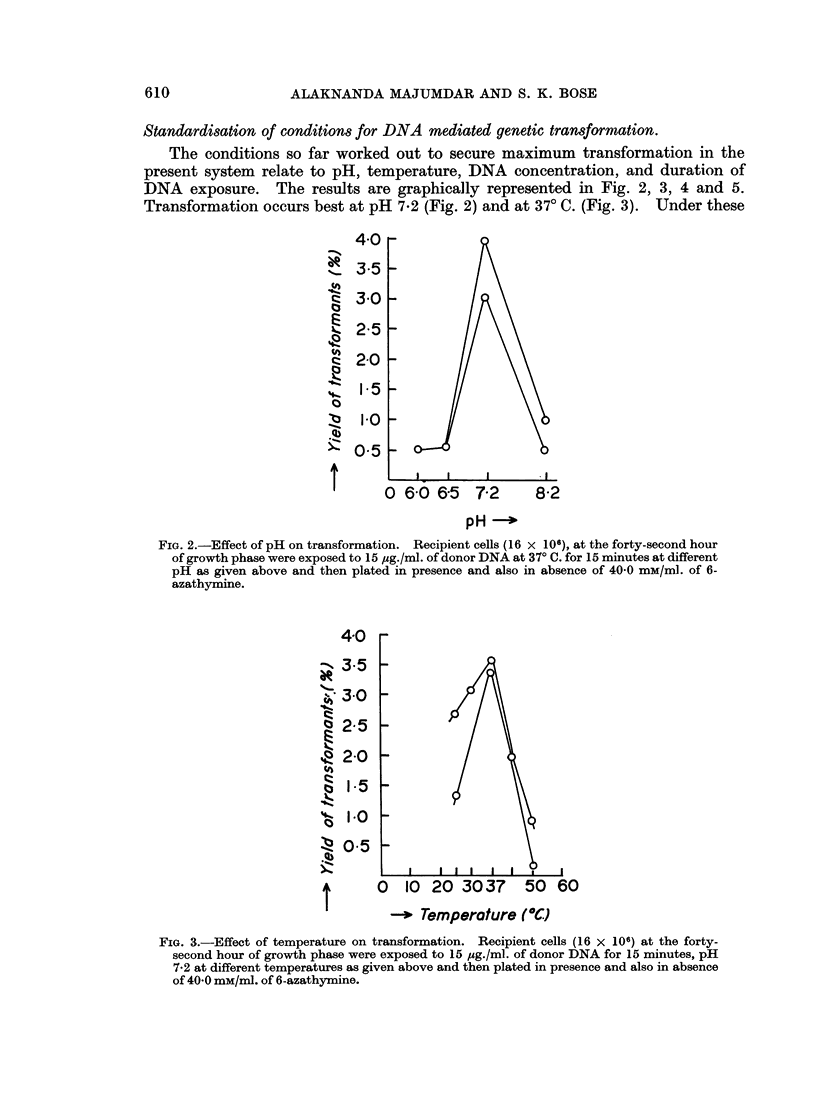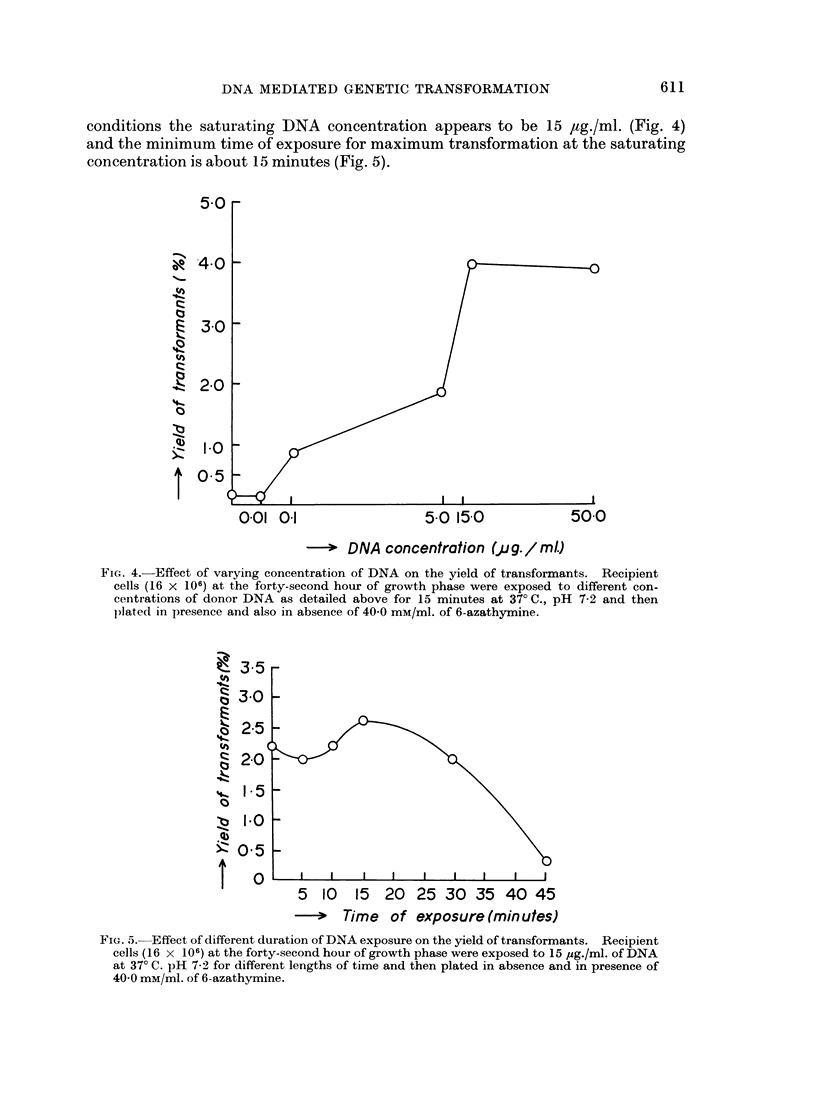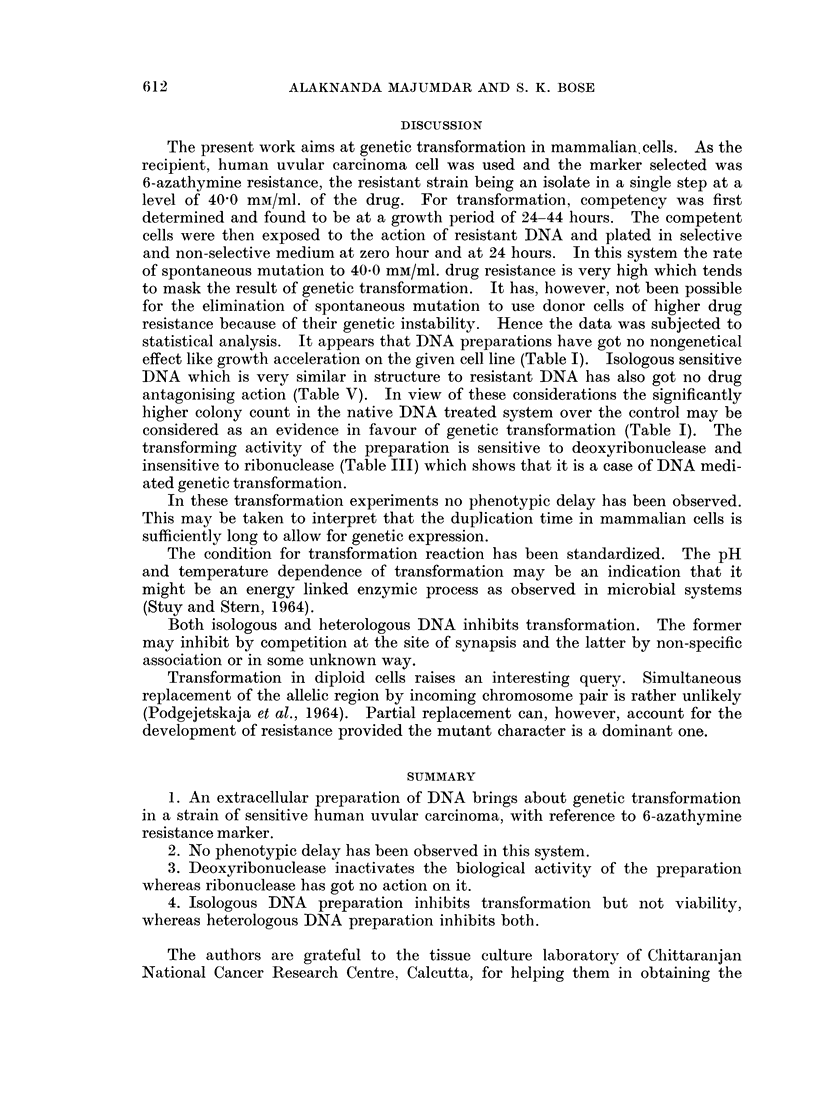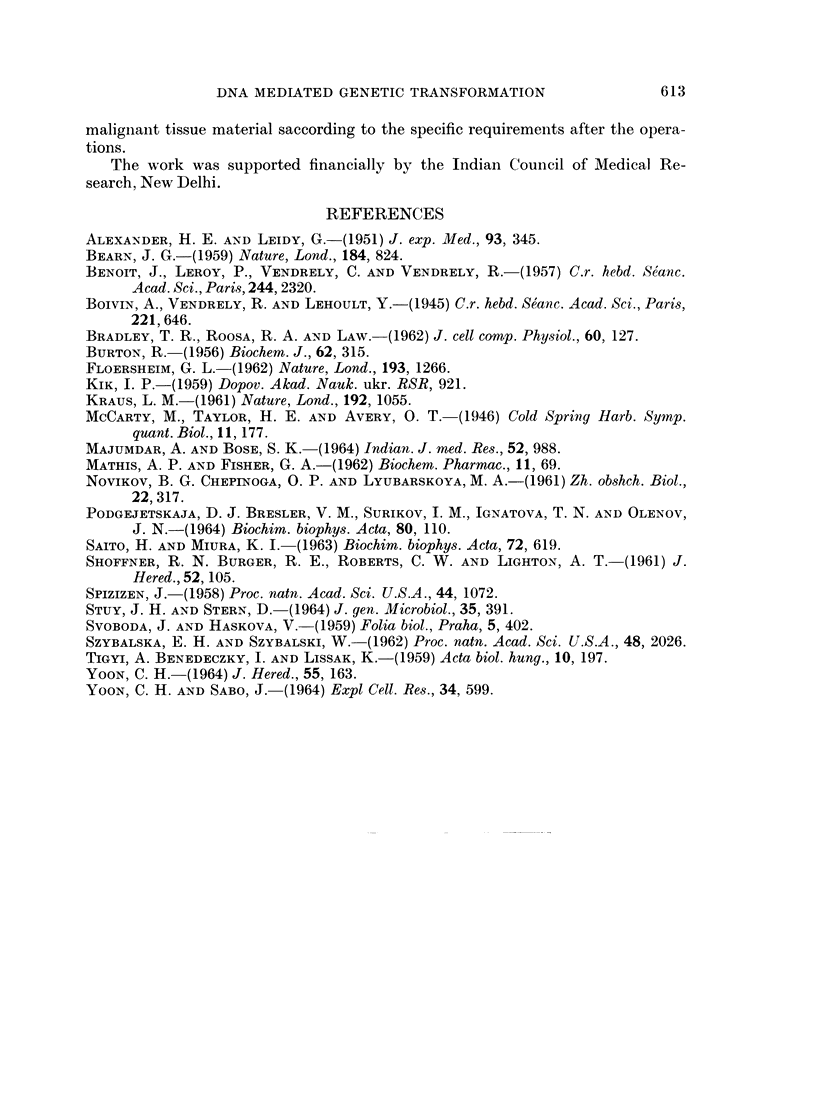# DNA mediated genetic transformation of a human cancerous cell line cultured in vitro.

**DOI:** 10.1038/bjc.1968.72

**Published:** 1968-09

**Authors:** A. Majumdar, S. K. Bose


### DNA MEDIATED GENETIC TRANSFORMATION OF A HUMAN CANCEROUS CELL LINE CULTURED *IN VITRO*

ALAKNANDA MAJUMDAR* AND S. K. BOSE

*From the Department of Biochemistry, University College of Sciences,* 35 *Ballygunge Circular Road, Calcutta*-19, *India*

Received for publication March 4, 1968

DNA MEDIATED genetic transformation is fairly established in many microbial systems including *Pneumococcus* (McCarty, Taylor and Avery, 1946), *B. subtilis* (Spizizen, 1958), *E. coli.* (Boivin, Vendrely and Lehoult, 1945), *Haemophilus* (Alexander and Leidy, 1951), etc. But in mammalian systems the problem has not been adequately explored. Results on transformation studies in intact animals are generally negative (Bearn, 1959; Kok, 1959; Shoffner *et al.*, 1961; Svoboda and Haskova, 1959; Tigyi, Benedeczky and Lissak, 1959). With ducks however some positive evidence has been recorded (Benoit *et al.*, 1957; Novikov, Chepinoga and Lyubarskoya, 1961). Genetic transformation has also been claimed in case of mammalian cells cultured both *in vitro* (Bradley *et al.*, 1962; Kraus, 1961; Szybalska and Szybalski, 1962) and *in vivo* (Podgejetskaja, 1964), although there are some cases of ambiguity in the interpretation of results on transformation (Bradley, Roosa and Law, 1962). This ambiguity or failure has so far been variously accounted for by the presence of insufficient number of recipient cells (Florsheim, 1962), high frequency of spontaneous mutation (Mathis and Fisher, 1962), the non-specific protective action of DNA (Bradley *et al.*, 1962) and lack of penetration of exogenous DNA (Yoon, 1964; Yoon and Sabo, 1964). That there are even cases in which the reported results could not be confirmed further complicate interpretation of data on mammalian transformation (Szybalska and Szybalski, 1962). In the present work a study has been made on DNA mediated genetic transformation of a clonal cell line of human uvular carcinoma isolated in this laboratory with reference to 6-azathymine (purchased from M/s. Sigma Chemical Company, U.S.A.) resistance marker.

### MATERIALS AND METHODS

### *Cell line*

A. Parental cell line is a clone obtained from a primary culture of human uvular carcinoma and subsequently maintained in liquid suspension culture.

B. Resistant cell line is an isolate in a single step at 40·0 mM/ml. level of 6-azathymine. In developing resistance, the sensitive cell line was cultivated in solid media (Majumdar and Bose, 1964). A single clonal isolate was then maintained in drug free liquid suspension culture.

* Present Address: Indian Institute of Experimental Medicine, Jadavpur, Calcutta-32, India.

*Media*

For liquid suspension culture the medium used was standard Eagle's basal medium supplemented with 150 ml. of normal horse serum, 10 units of penicillin G, and 0·5 g. of streptomycin base per litre.

For solid culture Eagle's basal medium containing 1 % washed agar was used.

*Isolation of DNA*

For isolation the modified phenol deproteinisation method of Kirby (Saito and Miura, 1963) was adopted. Resistant cell population grown in 1 litre medium for 72 hours at 37° C. in shake culture was harvested by centrifugation at 1000–1200 r.p.m. (0° C.) and washed once with saline-EDTA and resuspended in 6 ml. of saline-EDTA to which 50 ml. of Tris-sodium dodecyl sulphate solution was added. It was then frozen and thawed 4 times. Deproteinisation was done by equal volume of water saturated phenol (pH 9), and finally DNA precipitated by double volume of 95 % ethanol at 4° C. and dissolved in saline citrate solution (NaCl 0·15 M + $Na_3$-citrate 0·015 M). It was then dialysed overnight at 4° C. against the same saline citrate solution and stored at the same temperature. DNA was estimated by diphenyl amine method of Burton (1956).

*Labelling of DNA*

For labelling, cells were grown in bulk medium to which [$^{32}P_i$] $KH_2PO_4$ was added. And subsequently labelled DNA was isolated following the method described above.

*Technique of transformation*

In a typical transformation experiment $16 \times 10^6$ competent cells (42–44 hours of age), were washed once with balance salt solution (8·0 g. NaCl, 0·4 g. KCl, 0·35 g. $NaHCO_3$, 1·0 g. glucose per litre of water) and resuspended in phosphate buffered saline solution (7·0 g. NaCl, 0·4 g. KCl, 2·75 g. $Na_2HPO_4$, 0·25 g. $NaH_2PO_4$, 1·0 g. glucose per litre of water) containing 100 μg. spermine-HCl and 30 μg. donor DNA. The volume of the reaction mixture was adjusted to 2 ml. It was then incubated for 15 minutes at 37° C. Thereafter it was diluted hundred fold by non-selective basal medium and immediately plated in selective medium for 0 hour mutation. For phenotypic delay a definite aliquot of the diluted suspension was centrifuged, resuspended in the same volume of the non-selective medium overnight and plated as before.

### RESULTS

*Competency of the recipient population*

The competency of the recipient population was measured in terms of labelled DNA uptake (Fig. 1). In all, 3 parallel sets of experiments were performed using different DNA preparations having different specific activities. It appears that the uptake curves in case of hydrolysed DNA varies from preparation to preparation. With one preparation there is a continuous rise (Fig. 1, curve I B), with a second more than one peak (Fig. 1, curve II B) and with a third a single peak in the curve (Fig. 1, curve III B). In the uptake of native DNA all the 3 different preparations behave basically in the same way, in the sense that there is a single peak in the uptake, although a plateau (obviously due to insufficient data) has been observed in one case only (Fig. 1, curve I A). These peaks are again very sharp being at the forty second hour of cell growth. It may be taken to mean that the cells are competent only at the specific hour of growth and that competency persists only for a very short time in the generation cycle.

FIG. 1.—Competency of the sensitive recipient cells. Recipient cells (16 × $10^6$) harvested at different points of their growth phase (0–52 hours), were exposed to 15 μg./ml. of native and hydrolysed (by 1·5 μg./ml. of DNase in presence of $Mg^{++}$ions at 37° C. for 2 hours) $^{32}P$—DNA, at 37° C. for 15 minutes at pH 7·2. Cells after exposure were immediately centrifuged and washed repeatedly (usually for 4 times) with phosphate-buffered saline solution and then the pellet was plated in aluminium planchets and radioactive count taken in windowless gas flow counter (Tracer Lab. Inc., Sc. 16).

○————○, ○– – – – – –○, represent uptake curves of intact and hydrolysed DNA (specific activity 565·3), by sensitive recipient cells respectively. ■————■, ■– – – – – –■ and ●————●, ●– – – – – –●, represent the same results of two separate experiments with specific activity of donor $^{32}P$—DNA being 825·7 and 300·0 respectively.

*Transformation reaction*

Transformation reaction in the given system was performed with respect to 6-azathymine resistance marker, the donor DNA being obtained from the resistant cell line isolated at a concentration of 40·0 mM/ml. of the drug. Ten independent experiments were carried out using six different DNA preparations. The results are given in the Table I, and their statistical analysis in the Table II A and II B. It appears that DNA has no growth accelerating effect on the given cell line both at zero hour plating (Table II A, Column $A_1$ and $B_1$), and at 24 hours plating (Table II A, Column $A_2$ and $B_2$). The significantly higher colony count scored in selective medium in DNA treated system (Table I, Column $A_3$, $A_4$) over the control (Table I, Column $B_3$, $B_4$) may be taken as a positive evidence in favour of transformation (Table II A, Column $A_3$ and $B_3$, $A_4$ and $B_4$). The effect of

TABLE I.—*DNA-Mediated Genetic Transformation*

| No. of Experiments | System | Cells plated in absence of the drug (inoculum: $5 \times 10^2$ cells/plate) 0 hour plating | Cells plated in absence of the drug 24 hours plating | Cells plated in presence of 40·0 mM/ml. of 6-azathymine (inoculum: $1 \times 10^3$ cells/plate) 0 hour plating | Cells plated in presence of 40·0 mM/ml. of 6-azathymine 24 hours plating |
|---|---|---|---|---|---|
| | Native DNA | $A_1$ | $A_2$ | $A_3$ | $A_4$ |
| (i) | . . | 215 | 236 | 112 | 148 |
| (ii) | . . | 269 | 200 | 32 | 90 |
| (iii) | . . | 291 | 168 | 130 | 110 |
| (iv) | . . | 163 | 131 | 82 | 30 |
| (v) | . . | 324 | 195 | 67 | 20 |
| (vi) | . . | 223 | 237 | 19 | 90 |
| (vii) | . . | 236 | 240 | 41 | 30 |
| (viii) | . . | 246 | 236 | 17 | 19 |
| (ix) | . . | 200 | 281 | 45 | 10 |
| (x) | . . | 314 | 149 | 80 | 24 |
| (xi) | . . | 275 | 200 | 57 | 32 |
| | Control | $B_1$ | $B_2$ | $B_3$ | $B_4$ |
| (i) | . . | 172 | 292 | 21 | 75 |
| (ii) | . . | 184 | 235 | 16 | 108 |
| (iii) | . . | 240 | 113 | 92 | 25 |
| (iv) | . . | 245 | 132 | 80 | 27 |
| (v) | . . | 351 | 150 | 24 | 48 |
| (vi) | . . | 239 | 237 | 19 | 0 |
| (vii) | . . | 226 | 238 | 30 | 4 |
| (viii) | . . | 152 | 202 | 0 | 0 |
| (ix) | . . | 198 | 238 | 0 | 0 |
| (x) | . . | 267 | 133 | 3 | 18 |
| (xi) | . . | 260 | 100 | 3 | 19 |

$16 \times 10^6$ recipient cells were exposed at the forty-second hour of growth to 15 μg./ml. of resistant donor DNA for 15 minutes at 37° C. pH 7·2, diluted hundredfold and plated in absence of the drug and also in presence of 40·0 mM/ml. of 6-azathymine both at zero hour and at 24 hours. (Preincubation was done in non-selective medium.)

Results are given in terms of colony counts/plate.

TABLE II A.—*Statistical Analysis of Table* I

| Column | No. of experiments | t value | Conclusion |
|---|---|---|---|
| Comparison between figures with and without DNA | | | |
| $A_1$ and $B_1$ . . . . . . | 11 . | 0·731 . | insignificant* |
| $A_2$ and $B_2$ . . . . . . | 11 . | 0·641 . | insignificant* |
| $A_3$ and $B_3$ . . . . . . | 11 . | 4·31 . | significant at 1% level |
| $A_4$ and $B_4$ . . . . . . | 11 . | 3·180 . | significant at 1% level |
| Comparison between figures at 0 hour and 24 hours plating | | | |
| $B_3$ and $B_4$ . . . . . . | 11 . | 0·578 . | insignificant* |
| $A_3$ and $A_4$ . . . . . . | 11 . | 0·757 . | insignificant* |

* Insignificant at 5% level.

TABLE II B.—*Statistical Analysis of Table* I

| Comparison between figures with and without drug | t value | Conclusion |
|---|---|---|
| $\frac{A3}{A1} \longleftrightarrow \frac{B3}{B1}$ | 12·7429 | Significant at 1% level |
| $\frac{A4}{A2} \longleftrightarrow \frac{B4}{B2}$ | 8·9180 | Significant at 1% level |

phenotypic delay in transformation was also studied. In another set of parallel experiments the cells after DNA exposure were preincubated for 24 hours in non-selective medium and plated as before. The statistical analysis of the columns $B_3$ and $B_4$ in Table I indicates the absence of any increase in the value of spontaneous mutation (Table II A, $B_3$ and $B_4$). So the columns $A_3$ and $A_4$ of the Table I were directly analysed which shows that there is no phenotypic delay in the system (Table II A, $A_3$ and $A_4$).

It seems desirable on account of the large spread in plating efficiency of drug sensitive and resistant cells to compare statistically the percentage of cells plated in absence and in presence of the drug with and without DNA. The independent tests so carried out (Table II B) indicate the data to be significant at 1% level which supports the claim already made in favour of DNA mediated genetic transformation (Table II A).

*Effect of deoxyribonuclease and ribonuclease on the transforming activity of the preparation*

The effect of deoxyribonuclease and ribonuclease on the transforming activity of the preparation was studied (Table III) and the data statistically analysed (Table IV). It appears that native DNA, deoxyribonuclease treated or ribonuclease treated DNA has no growth accelerating effect on the cell line both at zero hour plating (Table IV, Columns A and B, C and B, D and B) and at 24 hours (Table IV, Columns $A_1$ and $B_1$, $C_1$ and $B_1$, $D_1$ and $B_1$). The colony counts recorded in column $A_2$ and $C_2$ are significantly higher thant those in $B_2$ in Table IV, which again supports transformation. No significant difference in the colony counts has been observed between $A_2$ and $D_2$ and $B_2$ and $C_2$, which indicates that the transforming activity of the preparation is ribonuclease insensitive but deoxyribonuclease sensitive.

On statistical analysis of the data of the columns $B_2$ and $B_3$ in Table IV, no significant difference in the count has been observed, which shows that there is no increase in the number of spontaneous mutants between zero hour and 24 hours plating. Consequently the data of the columns ($A_2$ and $A_3$, $C_2$ and $C_3$, $D_2$ and $D_3$) were directly compared statistically. Absence of any significant difference may be taken to mean that there is no phenotypic delay in genetic expression, as observed before.

*Effect of isologous and also unrelated DNA on the transforming activity of the preparation*

In this experiment standard L-strain, and sensitive parent strain were used as the source of heterologous and isologous DNA respectively. The effect was studied in 2 different concentrations (Table V). Isologous DNA does not affect, or does so very slightly, the colony count in non-selective medium. But in selective medium it adversely affects the count. Heterologous DNA on the other hand definitely decreases the colony count both in the non-selective and also in the selective medium. This may be taken to mean that the DNA preparations whether isologous or heterologous do not at any rate antagonise the action of the drug.

TABLE III.—*Effect of Deoxyribonuclease and Ribonuclease on Transforming Activity of the Preparation*

| System | Cells plated in absence of the drug (Inoculumn: 5 × $10^2$ cells/plate) | | | | Cells plated in presence of 40·0 mM/ml. of 6-azathymne (Inoculum: 1 × $10^3$ cells/plate) | | | |
|---|---|---|---|---|---|---|---|---|
| | 0 hr plating | | 24 hr plating | | 0 hr plating | | 24 hr plating | |
| Native DNA . . | A | 215, 269, 291, 163, 314, 275, 329 | $A_1$ | 236, 200, 168, 131, 149, 200, 195 | $A_2$ | 112, 32, 130, 82, 80, 57, 67 | $A_3$ | 148, 90, 110, 30, 24, 32, 30 |
| Control . . | B | 172, 184, 240, 245, 267, 260, 351 | $B_1$ | 292, 235, 113, 132, 133, 100, 150 | $B_2$ | 21, 16, 62, 40, 3, 3, 24 | $B_3$ | 75, 108, 25, 27, 18, 19, 27 |
| Deoxyribonuclease treated DNA* . | C | 200, 236, 245, 241, 274, 220, 317 | $C_1$ | 184, 187, 100, 103, 271, 285, 145 | $C_2$ | 6, 4, 93, 62, 10, 12, 30 | $C_3$ | 19, 5, 45, 60, 9, 14, 4 |
| Ribonuclease treated DNA† . | D | 200, 163, 225, 231, 271, 285, 268 | $D_1$ | 276, 200, 100, 82, 100, 110, 130 | $D_2$ | 58, 60, 102, 50, 65, 20, 70 | $D_3$ | 100, 90, 44, 80, 30, 25, 10 |

* 15 μg./ml. of donor DNA was treated with 1·5 μg./ml. of deoxyribonuclease (pancreas, B. grade, purchased from Calbiochem, U.S.A.) and $Mg^{++}$ion for 2 hours at 37° C.

† 15 μg./ml. of donor DNA was treated with 50 μg./ml. of ribonuclease (5 × crystallised, bovine pancreas, purchased from Sigma Chemical Company, U.S.A.) for 2 hours at 37° C.

16 × $10^6$ recipient cells were exposed at the forty-second hour of growth to native as well as enzyme treated DNA as detailed above for 15 minutes at 37° C., then plated in absence of the drug and also in presence of 40·0 mM/ml. of 6-azathymine, both at 0 hour and after 24 hours. (Pre-incubation was done in non-selective medium.)

Results are given in terms of colony counts/plate.

TABLE IV.—*Statistical Analysis of Table III*

| Column | No. of observations | t value | Conclusion |
|---|---|---|---|
| (1) B and A | 7 | 0·344 | Insignificant* |
| (2) $B_1$ and $A_1$ | 7 | 0·411 | ,, |
| (3) B and C | 7 | 0·036 | ,, |
| (4) $B_1$ and $C_1$ | 7 | 0·387 | ,, |
| (5) B and D | 7 | 0·648 | ,, |
| (6) $B_1$ and $D_1$ | 7 | 0·653 | ,, |
| (7) $B_2$ and $B_3$ | 7 | 1·15 | ,, |
| (8) $A_2$ and $A_3$ | 7 | 1·11 | ,, |
| (9) $C_2$ and $C_3$ | 7 | 0·955 | ,, |
| (10) $D_2$ and $D_3$ | 7 | 0·408 | ,, |
| (11) $B_2$ and $C_2$ | 7 | 0·726 | ,, |
| (12) $B_3$ and $C_3$ | 7 | 2·11 | Significant at 5% level |
| (13) $B_2$ and $D_2$ | 7 | 3·34 | Significant at 1% level |
| (14) $B_3$ and $D_3$ | 7 | 0·926 | Insignificant* |
| (15) $C_2$ and $D_2$ | 7 | 2·56 | Significant at 5% level |
| (16) $C_3$ and $D_3$ | 7 | 3·01 | Significant at 1% level |
| (17) $A_2$ and $D_2$ | 7 | 1·17 | Insignificant* |
| (18) $A_3$ and $D_3$ | 7 | 0·618 | ,, |
| (19) $A_2$ and $C_2$ | 7 | 3·39 | Significant at 1% level |
| (20) $A_3$ and $C_3$ | 7 | 3·21 | Significant at 1% level |
| (21) $A_2$ and $B_2$ | 7 | 4·01 | Significant at 1% level |
| (22) $A_3$ and $B_3$ | 7 | 1·48 | Insignificant* |

* Insignificant at 5% level.

TABLE V.—*Effect of Isologous and Heterologous DNA on Transformation*

| System | Non-selective medium* | | Selective medium† | |
|---|---|---|---|---|
| | Colony | Plating efficiency (%) | Colony | Yield of transformants (%) |
| 0 $\mu$gm./ml. resistant DNA | 186 | 41 | 54 | 6·0 |
| | 230 | | 68 | |
| 15 $\mu$g./ml resistant DNA | 238 | 40 | 82 | 8·2 |
| | 168 | | 83 | |
| | 200 | 32 | 66 | |
| 15 $\mu$g./ml. sensitive DNA | 128 | | 42 | 5·5 |
| 15 $\mu$g./ml. resistant DNA+ | 127 | 37 | 65 | |
| 15 $\mu$g./ml. sensitive DNA | 150 | | 45 | 5·4 |
| 50 $\mu$g./ml. sensitive DNA | 236 | 38 | 50 | 5·6 |
| | 193 | | 63 | |
| 15 $\mu$g./ml. resistant DNA+ | 150 | 36 | 18 | 2·3 |
| 50 $\mu$g./ml. sensitive DNA | 156 | | 28 | |
| 15 $\mu$g./ml. L-strain DNA | 184 | 28 | 16 | 1·5 |
| | 114 | | 20 | |
| 15 $\mu$g./ml. resistant DNA+ | 117 | 28 | 20 | 2·0 |
| 15 $\mu$g./ml. L-strain DNA | 120 | | 29 | |
| 50 $\mu$g./ml. L-strain DNA | 150 | 30 | 38 | 2·4 |
| | 200 | | 10 | |
| 15 $\mu$g./ml. resistant DNA+ | 159 | 31 | 35 | 3·6 |
| 50 $\mu$g./ml. L-strain DNA | 176 | | 38 | |

* No 6-azathymine and $5 \times 10^2$ cells/plate.
† 40·0 mM/ml. of 6-azathymine and $1 \times 10^3$ cells/plate.
Recipient cells exposed to varying DNA concentrations as detailed above at 37° C. for 15 minutes at pH 7·2.

*Standardisation of conditions for DNA mediated genetic transformation.*

The conditions so far worked out to secure maximum transformation in the present system relate to pH, temperature, DNA concentration, and duration of DNA exposure. The results are graphically represented in Fig. 2, 3, 4 and 5. Transformation occurs best at pH 7·2 (Fig. 2) and at 37° C. (Fig. 3). Under these

FIG. 2.—Effect of pH on transformation. Recipient cells (16 × $10^6$), at the forty-second hour of growth phase were exposed to 15 μg./ml. of donor DNA at 37° C. for 15 minutes at different pH as given above and then plated in presence and also in absence of 40·0 mM/ml. of 6-azathymine.

FIG. 3.—Effect of temperature on transformation. Recipient cells (16 × $10^6$) at the forty-second hour of growth phase were exposed to 15 μg./ml. of donor DNA for 15 minutes, pH 7·2 at different temperatures as given above and then plated in presence and also in absence of 40·0 mM/ml. of 6-azathymine.

conditions the saturating DNA concentration appears to be 15 μg./ml. (Fig. 4) and the minimum time of exposure for maximum transformation at the saturating concentration is about 15 minutes (Fig. 5).

FIG. 4.—Effect of varying concentration of DNA on the yield of transformants. Recipient cells (16 × $10^6$) at the forty-second hour of growth phase were exposed to different concentrations of donor DNA as detailed above for 15 minutes at 37° C., pH 7·2 and then plated in presence and also in absence of 40·0 mM/ml. of 6-azathymine.

FIG. 5.—Effect of different duration of DNA exposure on the yield of transformants. Recipient cells (16 × $10^6$) at the forty-second hour of growth phase were exposed to 15 μg./ml. of DNA at 37° C. pH 7·2 for different lengths of time and then plated in absence and in presence of 40·0 mM/ml. of 6-azathymine.

### DISCUSSION

The present work aims at genetic transformation in mammalian cells. As the recipient, human uvular carcinoma cell was used and the marker selected was 6-azathymine resistance, the resistant strain being an isolate in a single step at a level of 40·0 mM/ml. of the drug. For transformation, competency was first determined and found to be at a growth period of 24–44 hours. The competent cells were then exposed to the action of resistant DNA and plated in selective and non-selective medium at zero hour and at 24 hours. In this system the rate of spontaneous mutation to 40·0 mM/ml. drug resistance is very high which tends to mask the result of genetic transformation. It has, however, not been possible for the elimination of spontaneous mutation to use donor cells of higher drug resistance because of their genetic instability. Hence the data was subjected to statistical analysis. It appears that DNA preparations have got no nongenetical effect like growth acceleration on the given cell line (Table I). Isologous sensitive DNA which is very similar in structure to resistant DNA has also got no drug antagonising action (Table V). In view of these considerations the significantly higher colony count in the native DNA treated system over the control may be considered as an evidence in favour of genetic transformation (Table I). The transforming activity of the preparation is sensitive to deoxyribonuclease and insensitive to ribonuclease (Table III) which shows that it is a case of DNA mediated genetic transformation.

In these transformation experiments no phenotypic delay has been observed. This may be taken to interpret that the duplication time in mammalian cells is sufficiently long to allow for genetic expression.

The condition for transformation reaction has been standardized. The pH and temperature dependence of transformation may be an indication that it might be an energy linked enzymic process as observed in microbial systems (Stuy and Stern, 1964).

Both isologous and heterologous DNA inhibits transformation. The former may inhibit by competition at the site of synapsis and the latter by non-specific association or in some unknown way.

Transformation in diploid cells raises an interesting query. Simultaneous replacement of the allelic region by incoming chromosome pair is rather unlikely (Podgejetskaja *et al.*, 1964). Partial replacement can, however, account for the development of resistance provided the mutant character is a dominant one.

### SUMMARY

1. An extracellular preparation of DNA brings about genetic transformation in a strain of sensitive human uvular carcinoma, with reference to 6-azathymine resistance marker.

2. No phenotypic delay has been observed in this system.

3. Deoxyribonuclease inactivates the biological activity of the preparation whereas ribonuclease has got no action on it.

4. Isologous DNA preparation inhibits transformation but not viability, whereas heterologous DNA preparation inhibits both.

The authors are grateful to the tissue culture laboratory of Chittaranjan National Cancer Research Centre, Calcutta, for helping them in obtaining the malignant tissue material saccording to the specific requirements after the operations.

The work was supported financially by the Indian Council of Medical Research, New Delhi.

### REFERENCES

ALEXANDER, H. E. AND LEIDY, G.—(1951) *J. exp. Med.*, **93**, 345.
BEARN, J. G.—(1959) *Nature, Lond.*, **184**, 824.
BENOIT, J., LEROY, P., VENDRELY, C. AND VENDRELY, R.—(1957) *C.r. hebd. Séanc. Acad. Sci., Paris*, **244**, 2320.
BOIVIN, A., VENDRELY, R. AND LEHOULT, Y.—(1945) *C.r. hebd. Séanc. Acad. Sci., Paris*, **221**, 646.
BRADLEY, T. R., ROOSA, R. A. AND LAW.—(1962) *J. cell comp. Physiol.*, **60**, 127.
BURTON, R.—(1956) *Biochem. J.*, **62**, 315.
FLOERSHEIM, G. L.—(1962) *Nature, Lond.*, **193**, 1266.
KIK, I. P.—(1959) *Dopov. Akad. Nauk.* ukr. *RSR*, 921.
KRAUS, L. M.—(1961) *Nature, Lond.*, **192**, 1055.
McCARTY, M., TAYLOR, H. E. AND AVERY, O. T.—(1946) *Cold Spring Harb. Symp. quant. Biol.*, **11**, 177.
MAJUMDAR, A. AND BOSE, S. K.—(1964) *Indian. J. med. Res.*, **52**, 988.
MATHIS, A. P. AND FISHER, G. A.—(1962) *Biochem. Pharmac.*, **11**, 69.
NOVIKOV, B. G. CHEPINOGA, O. P. AND LYUBARSKOYA, M. A.—(1961) *Zh. obshch. Biol.*, **22**, 317.
PODGEJETSKAJA, D. J. BRESLER, V. M., SURIKOV, I. M., IGNATOVA, T. N. AND OLENOV, J. N.—(1964) *Biochim. biophys. Acta*, **80**, 110.
SAITO, H. AND MIURA, K. I.—(1963) *Biochim. biophys. Acta*, **72**, 619.
SHOFFNER, R. N. BURGER, R. E., ROBERTS, C. W. AND LIGHTON, A. T.—(1961) *J. Hered.*, **52**, 105.
SPIZIZEN, J.—(1958) *Proc. natn. Acad. Sci. U.S.A.*, **44**, 1072.
STUY, J. H. AND STERN, D.—(1964) *J. gen. Microbiol.*, **35**, 391.
SVOBODA, J. AND HASKOVA, V.—(1959) *Folia biol., Praha*, **5**, 402.
SZYBALSKA, E. H. AND SZYBALSKI, W.—(1962) *Proc. natn. Acad. Sci. U.S.A.*, **48**, 2026.
TIGYI, A. BENEDECZKY, I. AND LISSAK, K.—(1959) *Acta biol. hung.*, **10**, 197.
YOON, C. H.—(1964) *J. Hered.*, **55**, 163.
YOON, C. H. AND SABO, J.—(1964) *Expl Cell. Res.*, **34**, 599.